# A Good App Is Hard to Find: Examining Differences in Racialized Sexual Discrimination across Online Intimate Partner-Seeking Venues

**DOI:** 10.3390/ijerph19148727

**Published:** 2022-07-18

**Authors:** Ryan M. Wade, Matthew M. Pear

**Affiliations:** School of Social Work, University of Illinois Urbana-Champaign, Urbana, IL 61801, USA; pear2@illinois.edu

**Keywords:** sexual racism, discrimination, gay/bisexual men, mobile apps, online dating

## Abstract

Young sexual minority Black men (YSMBM) report widespread instances of Racialized Sexual Discrimination (RSD) when seeking intimate partners online. RSD is associated with negative psychological health outcomes; however, little is known about the differences between virtual environments, and whether users are exposed to differential types/frequencies of RSD across different virtual environments. Using data from a cross-sectional web survey of YSMBM (*N* = 548), a multivariate Kruskal–Wallis test was conducted comparing those who primarily used Jack’d and those who primarily used Grindr to meet intimate partners; the frequency with which these two groups encountered six RSD domains was compared. Men who primarily used Grindr reported more frequent instances of White superiority and rejection from White men compared with men who primarily used Jack’d. Men who primarily used Jack’d reported more frequent instances of physical objectification from Black men compared with men who primarily used Grindr. RSD may manifest differentially based on the specific venue that YSMBM use. Such differences may reflect the sociodemographic makeup of these spaces, as well as differences in acceptability/normalization of different forms of RSD. These findings have implications for the development of anti-RSD initiatives that target the specific sociocultural norms that are unique to different virtual environments.

## 1. Introduction

The use of the internet for dating has become increasingly common in the United States, particularly for LGBTQ+ populations, who report considerably more frequent usage of dating apps and websites compared with their heterosexual counterparts [1,2,3]. LGBTQ+ persons have long embraced internet technology for social and sexual networking to circumvent discrimination and homophobia [4,5,6,7]. For sexual minority men, researchers believe that these virtual environments are important, as they offer a sense of safety to partner selection and can allow sexual minority men to connect with one another, even in predominantly straight spaces [8,9].

Virtual spaces that facilitate intimate partner-seeking have a number of unique and often critical disadvantages. The greater anonymity of virtual environments enables users to express harmful and discriminatory sentiments much more visibly, frequently, and aggressively than in most physical environments [10,11,12,13,14,15]. Discrimination is particularly explicit in these spaces, where users often discuss racialized people as non-preferred “types,” using phrases such as “no femmes/Asians” and “not into Black guys [16,17,18,19]. The valuing of “Whiteness” also predominates in these spaces. Whiteness embodies the notion that certain individuals (typically, those with fair skin and Eurocentric features) represent a normative, ideal standard to which other (non-White) groups are juxtaposed, ‘othered,’ and systematically disenfranchised [17,20,21]. Whiteness or Eurocentric features are regarded as the most desirable characteristics in an intimate partner and are often sought after by both White men and men of color alike [22,23,24,25]. Men of color also find that their messages are frequently ignored by other users and that the messages they receive often reject them on racialist grounds [26,27,28,29].

An exception to this systemic exclusion is the erotic objectification of men of color. Patterns of objectification draw on sexual scripts that typecast certain racial/ethnic groups into specific sexual roles or fetishize certain groups on the basis of perceived physical and behavioral characteristics [10,30,31,32]. In the case of young sexual minority Black men (YSMBM), they are often stereotyped as being sexually dominant, typically the insertive partner during anal sex, and as having large penises [31,33,34]. As race has no basis in biology, various researchers have argued that this discrimination—commonly described as sexual racism—is inextricable from broader patterns of White supremacy [27,35,36].

Given the prevalence of dating app use among YSMBM, their mental health may be at risk due to discrimination experienced on these platforms. A growing body of research examines the associations between this discrimination, described here as Racialized Sexual Discrimination (RSD), and health outcomes for men of color who encounter it. RSD is a multidimensional construct that describes the sexualized discriminatory treatment that sexual minority men of color encounter in online dating environments [37]. Researchers have reported that various forms of RSD may be associated with negative psychological symptoms such as lower self-esteem, depression, anxiety, suicidal ideation, disordered eating, and self-identified stress [38,39,40,41,42,43,44]. In a recent study, we reported on the association between multiple distinct manifestations of RSD and psychological wellbeing, using factors derived from the first psychometrically evaluated scale of RSD [45]. Among a large sample (*N* = 603) of YSMBM, White superiority (e.g., the elevation of Whiteness as the most desirable characteristic in an intimate partner), rejection from Black men, and physical objectification from White men were all significantly associated with an increase in self-reported depressive symptoms. White physical objectification was also associated with a decrease in self-reported feelings of self-worth among the study sample.

Relatively little is known about the differences between virtual environments in the prevalence of RSD. There are a variety of online intimate partner-seeking platforms for YSMBM, each with its own policies and practices for regulating RSD and each with its own set of user demographics; this suggests that experiences of RSD may therefore differ by platform. To date, little research has been conducted to examine the differences between platforms in how YSMBM experience RSD. In a study focused on comparing the stigma between YSMBM app users and non-users, Rosengren et al. (2019) analyzed correlations between users of various apps and several scales of general discrimination. The authors reported that Jack’d users scored lower than average in experienced racial discrimination; however, they did not specifically examine RSD.

The present study used the aforementioned RSD scale to examine the experiences of YSMBM who primarily use Grindr versus those who primarily use Jack’d. Neither app uses a matching algorithm, instead presenting profiles of potential partners based only on location and user-selected filters. Both apps also provide a high degree of anonymity to users who can establish accounts without verified identities or pictures of their faces. Some researchers have suggested that this anonymity may exacerbate the frequency and severity of RSD on these apps [10,22,31,46].

The apps have distinct user bases and reputations regarding the diversity of their users and the treatment of men of color on their platforms [47]. Despite Grindr having more overall users, researchers have found Jack’d to be more popular among YSMBM [1,48,49]. Jack’d touts its own diversity and inclusiveness, with its marketing director claiming in 2017 that its user base is 30% Black, 25% Asian, and 25% Latino/mixed/other and that staff and leadership were diverse as well [50,51]. Grindr, on the other hand, is commonly described in the press as a White-dominated environment in which White men frequently exclude or objectify men of color [52,53,54]. Grindr was threatened with a heavily publicized 2018 lawsuit over RSD [55] and Jack’d executives have repeatedly accused Grindr of not enforcing prohibitions on racist language [50]. Popular LGBTQ+ news outlets have also suggested that the culture of Jack’d is more affirming of men of color and that the sociodemographic makeup of Jack’d is considerably more diverse than other apps, including Grindr [56,57]. While these narratives are widespread, empirical investigation of these claims is very much in its infancy. Careful examination of the degree to which RSD is perpetuated across these virtual environments may have important implications for the health of sexual minority men of color.

User demographics, reputations, and anecdotal reports may not be reliable indicators of the prevalence of RSD on a given platform. The positive reputation of Jack’d does not necessarily imply that men of color using Jack’d experience less RSD than they would encounter on other platforms, as reputations are, in part, generated by marketing. The public scrutiny of Grindr does not necessarily imply that RSD is more prevalent there than on other platforms, as its greater size warrants greater public scrutiny. Furthermore, the relatively diverse user base of Jack’d does not guarantee a lack of RSD, as users who objectify Black men may be more likely to use a platform that has a large user base of Black men. Because of these considerations, the prevalence of various kinds of RSD on Jack’d and Grindr is an empirical question, and little social science research has yet been conducted to compare the prevalence of experiences of RSD amongst users on each app.

Given the importance of RSD and the paucity of research on this topic, this study aims to examine the frequency with which YSMBM encounter RSD, depending on which app (Grindr or Jack’d) they primarily use. Considering the general discourse surrounding the purported cultural and demographic characteristics of these venues, we predict that participants who primarily use Grindr will report higher frequencies of encountering White superiority, White rejection, White physical objectification, and sexual role assumptions compared with participants who primarily use Jack’d. We predict that participants who primarily use Jack’d will report higher frequencies of encountering same-race rejection and same-race physical objectification compared with participants who primarily use Grindr.

## 2. Materials and Methods

### 2.1. Participants

*Eligibility criteria***:** Participants had to meet the following eligibility criteria: (1) identify as a man; (2) be assigned male sex at birth; (3) identify primarily as Black, African American, or with any other racial/ethnic identity across the African diaspora (e.g., Afro-Caribbean, African, etc.); (4) be between the ages of 18 and 29 inclusive; (5) identify as gay, bisexual, queer, same-gender-loving, or another non-heterosexual identity, or report having had any sexual contact with a man in the last 3 months; (6) report having used a website or mobile app to find male partners for sexual activity in the last 3 months; and (7) reside in the United States.

### 2.2. Recruitment, Screening, and Consent

A non-probability convenience sample of YSMBM were recruited using best practices for online survey sampling [58,59] between July 2017 and January 2018. Participants were recruited online to participate in the “ProfileD Study”. Most participants were recruited through Facebook (*n* = 91.8%) and Scruff = (*n* = 5.7%). Study advertisements were hosted on each platform. Prospective participants clicked on a link embedded in the advertisement that directed them to the study webpage hosted on Qualtrics.

Upon arriving at the study webpage, participants completed a set of screening questions to determine their eligibility. Participants responded to a series of yes or no questions about their gender, age, racial/ethnic identity, sexual orientation/sexual behavior, mobile app or website use, and residence. Prospective participants who met the eligibility criteria and completed the screening form were brought to a consent page, which contained detailed study information (i.e., purpose of the research, description of participant involvement, risk/discomforts; benefits; confidentiality etc.). Those consenting to participate proceeded to the full survey.

### 2.3. Procedure

Those consenting to participate in the study completed a survey on Qualtrics lasting 30 to 45 min. Participants were not compensated for taking the survey. While completing the survey, participants were permitted to save their answers and return to the survey at a later time if they were not able to complete it in a single sitting. Study data were kept in an encrypted and firewall-protected server, and the Institutional Review Board at (University of Michigan) approved all study procedures.

### 2.4. Measures

#### 2.4.1. Sociodemographics

The self-reported age, educational attainment, and sexual orientation of each participant was collected for descriptive purposes. Participants were instructed to provide their numerical age. Participants could select one of 11 sexual orientation categories: 1 = ‘Gay’; 2 = ‘Bisexual’; 3 = ‘Same Gender Loving’; 4 = ‘Queer’; 5 = ‘Straight’; 6 = ‘Trade’; 7 = ‘Down Low (DL)’; 8 = ‘Homothug’; 9 = ‘Questioning’; 10 = ‘Other’; and 11 = ‘Unsure’. Educational attainment was measured using a 5-point Likert scale (1 = ‘less than high school’; 2 = ‘high school graduate’; 3 = ‘some college’; 4 = ‘college graduate’; and 5 = ‘post college’). Finally, participants were provided with a list of 18 different dating/hook-up websites or mobile apps catered towards gay/bisexual men, and were asked to indicate which app they primarily used to seek intimate partners.

#### 2.4.2. Racialized Sexual Discrimination

Data were collected on participants’ self-reported experiences of RSD using the Racialized Sexual Discrimination Scale (RSDS) [60]. The RSDS is a multidimensional measure of the phenomenon that assesses different forms of RSD across multiple contexts, while accounting for the frequency and effect of racial experiences, and the race of the users perpetuating RSD. For the present study, the frequency of RSD experiences across six psychometrically validated subscales was measured. Experiences described on the scale could occur in one of two contexts: partner browsing (i.e., viewing user profiles on mobile apps/websites) and partner negotiation (i.e., written communication between users on mobile apps/websites). Items within the partner browsing context were measured on a 5-point Likert scale (0 = ‘never’; 1 = ‘some of the time’; 2 = ‘half of the time’; 3 = ‘most of the time’; and 4 = ‘all of the time’). Items within the partner negotiation context were measured on a 6-point Likert scale (0 = ‘I have not contacted this group’; 1 = ‘never’; 2 = ‘some of the time’; 3 = ‘half of the time’; 4 = ‘most of the time’; and 5 = ‘all of the time’) items. For ease of interpretation, each individual item within the partner browsing context was divided by four and multiplied by 100, and each item within the partner browsing context was divided by five and multiplied by 100. Thus, all items on the scale were scored from 0 to 100 for data analysis.

#### 2.4.3. White Superiority and Role Assumptions

The White superiority subscale score was computed using the mean of eight items (e.g., ‘How often do you see profiles from White people clearly state that they want to meet other White people (partner browsing)’; ‘How often do White people say something mean or hurtful about your race/ethnicity (partner negotiation)?’). The role assumptions subscale was computed using the mean of six items (e.g., ‘How often do you see profiles from White people assume that people of your race/ethnicity will take on a particular sexual role (partner browsing)’; ‘How often do people of your race/ethnicity assume that you will take on a particular sexual role because of your race/ethnicity (partner negotiation)?’). The Cronbach’s alpha value for White superiority (α = 0.823) and role assumptions (α = 0.833) demonstrated strong reliability.

#### 2.4.4. Rejection

The White rejection subscale score was computed using the mean of two items (‘How often are your messages rejected by White people’; ‘How often are your messages ignored by White people?’). The same-race rejection subscale score was computed using the mean of two items (‘How often are your messages rejected by people of your race/ethnicity’; ‘How often are your messages ignored by people of your race/ethnicity’). The Cronbach’s alpha value for White rejection (α = 0.931) and same-race rejection (α = 0.886) demonstrated strong to excellent reliability.

#### 2.4.5. Physical Objectification

The White physical objectification subscale score was computed using the mean of two items (‘How often do you see profiles from White people express a desire for a specific physical trait related to people of your race/ethnicity (partner browsing)’; ‘How often do White people express a desire for a specific physical trait related to your race/ethnicity (partner negotiation)?’). The same-race physical objectification subscale score was computed using the mean of two items (‘How often do you see profiles from people of your race/ethnicity express a desire for a specific physical trait related to other people of your race/ethnicity (partner browsing)’; ‘How often do people of your race/ethnicity express a desire for a specific physical trait related to your race/ethnicity (partner negotiation)?’). The Cronbach’s alpha value for White physical objectification (α = 0.801) and same-race physical objectification (α = 0.772) demonstrated acceptable to strong reliability.

### 2.5. Data Collection and Cleaning

A total of 2,188 eligible and consenting participants were recruited for the study. Participants who did not provide any information on their preferred app (*n* = 257) could not be analyzed and were thus excluded. Due to insufficient sample sizes across the other 16 mobile apps/websites, participants who primarily used an app/website other than Grindr or Jack’d (*n* = 457) were also excluded from analysis. Finally, participants who dropped out of the survey before completing the RSD scale were excluded (*n* = 926). Thus, our final analytic sample consisted of 548 participants, with 381 participants identifying Grindr as their primary app (69.5%) and 167 participants identifying Jack’d as their primary app (30.5%).

### 2.6. Data Analytic Strategy

Descriptive statistics were computed for the study sample, including mean scores, frequency counts, and percentages for demographic characteristics and study variables. To explore differences between virtual venues, a multivariate Kruskal–Wallis (MKW) test was conducted comparing two groups: (1) those who primarily used Jack’d to meet partners and (2) those who primarily used Grindr to meet partners, and the frequency with which these two groups encountered six RSD domains was compared. To reduce the risk of encountering a type I error, a significance value of *p* < 0.01 was selected as the minimum value to establish statistical significance. The significance value was reported using the Kruskal–Wallis *H* statistic (chi-square approximation), and mean ranks are reported for all outcomes across each group. Bivariate associations between these six RSD domains were also computed, and all data were analyzed using SPSS v. 20.

## 3. Results

### 3.1. Sample Description

The mean age of the sample was 24.16 years (*SD* = 3.15). Most participants identified as gay (70.6%) or bisexual (16.2%). The sample was well-educated, as nearly half (44.4%) of participants had completed a college degree and/or received a post-graduate education. The other half had mostly received some college education (43.1%), and only one participant had not completed high school. White rejection (*M* = 57.50) and White objectification (*M* = 54.36) were the most frequently occurring manifestations of RSD reported among the study sample overall. Same-race objectification (*M* = 45.63) was comparatively lower, and same-race rejection (*M* = 42.52), White superiority (*M* = 42.02), and role assumptions (*M* = 41.70) were the least frequently occurring manifestations of RSD (see Table 1). The majority (97.6%) of participants reported using more than one app/website to find partners, with 74.9% of primary Jack’d users indicating that they also used Grindr, and 53.5% of primary Grindr users indicating that they also used Jack’d. Notably, 99.8% of the study sample reported encountering at least one instance of one of the six RSD domains. Correlations among subscales ranged from 0.11 (White rejection and White physical objectification) to 0.48 (White superiority and role assumptions). All subscales were significantly correlated with one another at the 0.05 level or below (see Table 2).

### 3.2. Analysis of Variance

The MKW test showed that there was a statistically significant difference between the two groups on three out of the six RSD frequency domains. Men who primarily used Grindr (mean rank = 290.15) reported more frequent instances of White superiority (*H*_(1)_ = 12.22, *p* < 0.001) compared with men who primarily used Jack’d (mean rank = 238.79). Men who primarily used Grindr (mean rank = 304.87) also reported more frequent instances of rejection from White men (*H*_(1)_ = 47.85, *p* < 0.001) compared with men who primarily used Jack’d (mean rank = 205.79). Men who primarily used Jack’d (mean rank = 336.37) reported more frequent instances of physical objectification from Black men (*H*_(1)_ = 38.00, *p* < 0.001) compared with men who primarily used Grindr (mean rank = 247.38) (see Table 3).

## 4. Discussion

**Figure 1:**
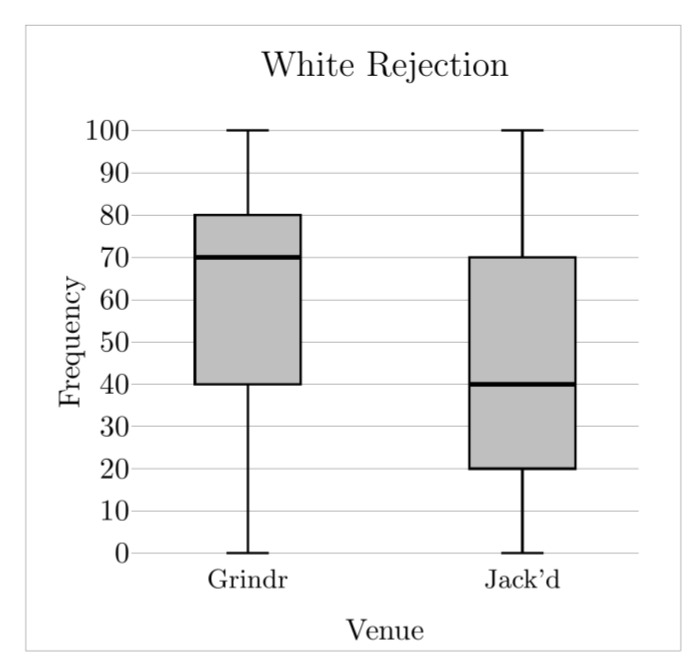
White rejection frequency across venues. Middle solid line indicates the median value.

**Figure 2:**
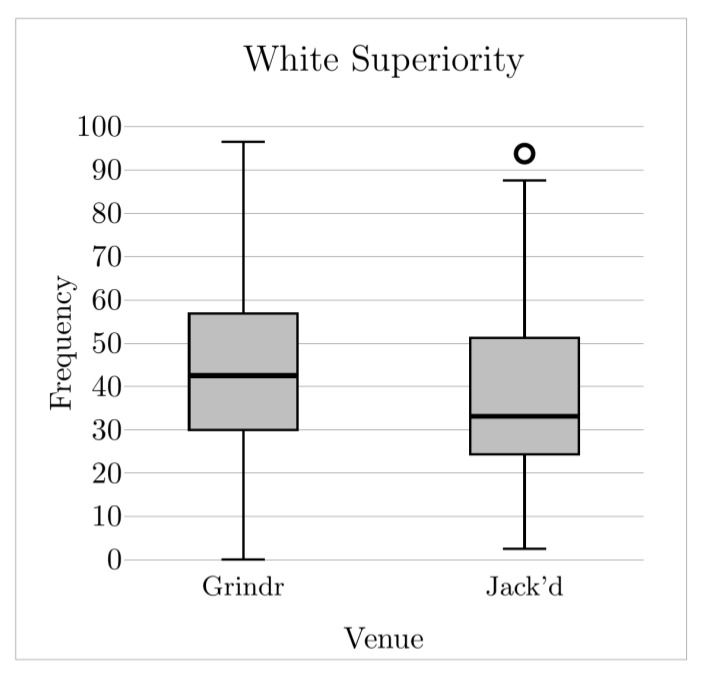
White superiority frequency across venues. Middle solid line indicates the median value; circles indicate outliers.

**Figure 3:**
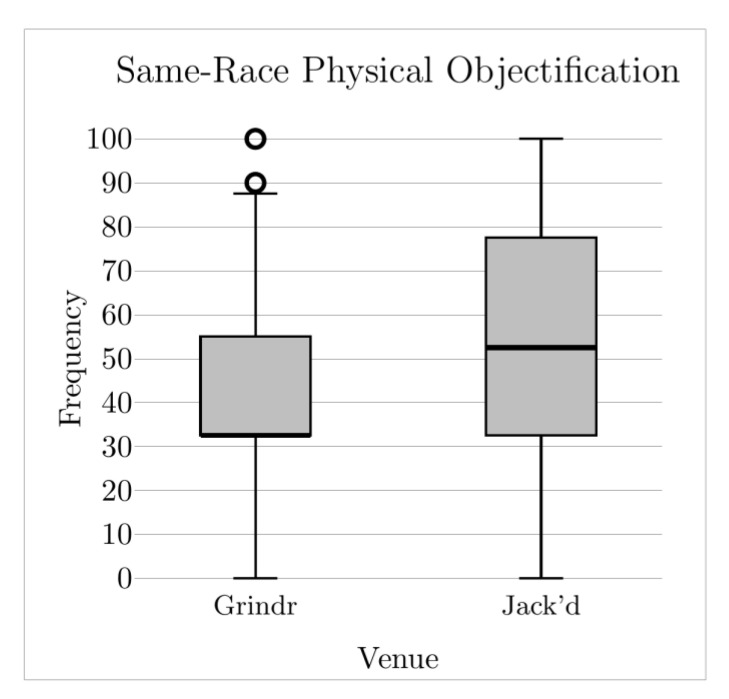
Same-race physical objectification frequency across venues. Middle solid line indicates the median value; circles indicate outliers.

This study compared the frequency with which six distinct RSD domains were reported between YSMBM who primarily used Grindr, and YSMBM who primarily used Jack’d. Frequencies were overall high among the entire study sample. Among participants who primarily used Grindr, White rejection was the most frequently occurring manifestation of RSD by a considerable margin. Among participants who primarily used Jack’d, White and same-race physical objectification were the most frequently occurring manifestations of RSD. Our prediction that participants who primarily used Grindr would report higher frequencies of encountering White rejection (Figure 1) and White superiority (Figure 2) was confirmed, as was our prediction that participants who primarily used Jack’d would report higher frequencies of same-race physical objectification (Figure 3). Contrary to predictions, we found no differences on White physical objectification, same-race rejection, or role assumptions frequencies based on primary venue.

Our findings that White superiority and White rejection were more frequently encountered among users who primarily used Grindr is consistent with reports of Grindr’s sociocultural norms, particularly with respect to the propensity of many White users to systematically exclude men of color, and the tendency for users to position Whiteness as the most desired characteristic in an intimate partner [52,53,54]. It may also be reflective of a higher proportion of White users on Grindr, such that the likelihood of exposure to these particular forms of RSD may be higher on this platform. Similarly, our findings that same-race physical objectification was more frequently encountered among users who primarily used Jack’d may also be reflective of the sociodemographic makeup of the Jack’d user base, which purportedly consists of a higher proportion of YSMBM relative to Grindr and other apps [1,48,49].

Our findings that same-race rejection was not more frequently reported on Jack’d, though contrary to hypotheses, may also be illuminating about the nature of different virtual venues and the individuals who inhabit them. If rejection from other Black users does not occur significantly more frequently on Jack’d than on Grindr, in spite of the purportedly higher number of Black users on Jack’d, this may indicate that YSMBM are more sought after on Jack’d in relative terms, based on the sociodemographic differences of these two dating environments. This may be particularly important in light of our previous research, in which we found that same-race rejection was significantly associated with higher self-reported depressive symptomatology among YSMBM [45]. In this same study, we found that White superiority was also associated with higher scores on depressive symptoms, and our current study findings indicate that White superiority is encountered much more frequently on Grindr compared with Jack’d. In addition, although same-race physical objectification occurred more frequently on Jack’d compared with Grindr, same-race physical objectification was not associated with negative mental health outcomes in our prior work. Taken together, these findings lend credence to the idea that some virtual environments, such as Jack’d, may be less harmful than others, though they may not be entirely devoid of RSD.

## 5. Implications

The high frequency of RSD reported overall by primary users of both Grindr and Jack’d suggests that RSD pervades both environments and may pervade similar environments as well. This suggests the necessity of addressing RSD in all online dating environments by mitigating its impact and disrupting its practice. On the other hand, the disparity in frequencies of various forms of RSD reported by primary users of Jack’d and Grindr suggests that different forms of RSD are not equally pervasive in all online dating environments. This indicates that distinctions between environments may influence how interventions are prioritized and designed in broadly addressing RSD.

Awareness initiatives may be helpful in discouraging app users from engaging in RSD and in equipping mental health practitioners and the targets of RSD to mitigate its effects. Such initiatives can impart information regarding not only the overall prevalence and impact of RSD, but also its pervasiveness in different environments. This messaging might sometimes take the form of advertisements on dating platforms, which would be tailored to the demographics and common experiences of users on a particular app. Interventions might also take the form of organizers enlisting app users who disapprove of RSD to oppose it publicly and effectively within the virtual environment itself. This can involve encouragement to use anti-racist messaging in profiles, to confront and report those with racist profiles, and to assertively address racist personal messages. Such a campaign would include guidance for how to perform this effectively and could even provide copy-and-paste templates to make intervention faster and less stressful for user-activists.

The findings of this study suggest that targeting such interventions at specific environments and tailoring interventions to user demographics and app culture is important. A collaborative effort between researchers and app administrators to influence the design of online environments could be effective in combatting RSD. While administrators have access to extensive information on user actions, they may not be aware of the psychological impacts of various forms of RSD or of the particular forms of RSD that are disproportionately prevalent on their platforms. Some app administrators are in a better position than others to proactively address RSD on their platforms. Scruff, which does not rely on outside investment, discontinued third-party advertisements on their platform in 2018. They claimed that short-term profits suffered because of the move, but that they could afford to be more far-sighted than competitors who rely on external funding [61]. Scruff acquired Jack’d in 2019 and discontinued third-party advertising there as well [62]. The owners of Scruff and Jack’d provide one example of app administrators who can potentially spearhead collaboration with researchers to address RSD on their platforms in the interest of long-term profitability and perhaps personal ideology. Such administrators are still ultimately constrained by their need for revenue, but they have more leeway than competitors who are beholden to investors.

Researchers, activists, and tech entrepreneurs can also collaborate to establish alternative virtual spaces for sexual minority men who oppose all facets of RSD. These spaces can serve as a way for anti-racist sexual minority men to network with one another more efficiently and circumvent the discrimination that is commonplace in other virtual environments. This would provide a space for men to discuss their experiences, share advice and encouragement, and provide one another with emotional support. Such a space would not only serve to mitigate the effects of RSD for sexual minority men of color, but could also foster the development of campaigns against RSD through facilitating the recruitment and support of activists. Virtual environments designed with the express intention of subverting or countering discrimination may prove to be an invaluable and necessary step towards combatting RSD and promoting collective resilience among sexual minority men.

## 6. Strengths and Limitations

The study has several important limitations. First, the cross-sectional nature of the data and our non-representative sample limits the generalizability of our findings. Moreover, given that the study was lengthy and unpaid, many participants opted not to complete it in full. While our final analytic sample provided more than sufficient power to test our research questions, the participants who dropped out of the survey early may systematically differ from those who finished it (e.g., these participants may have had significantly higher or significantly lower scores on the study outcomes under investigation). Future research should introduce additional safeguards to maximize participant retention.

The analysis presented in this study is also based on associations between participants’ primary online dating app or website (in this case, the apps Jack’d and Grindr). As a result, we are unable to make inferences about the frequency with which users experience RSD on other platforms. In addition, most study participants reported using sites and apps other than their primary app; thus, some reported instances of RSD may have taken place in non-primary environments. It is possible, too, that some participants may favor their primary app in order to avoid harmful forms of RSD that they experience disproportionately in non-primary environments. There is emerging empirical support for this possibility, as investigators have reported that sexual minority Black men may seek intimate encounters exclusively with other Black men in an effort to avoid racial discrimination [40,63]. Thus, YSMBM who are repeatedly exposed to RSD may seek out alternative virtual environments where they are less likely to encounter discrimination. Researchers should therefore investigate the motivations driving participants’ app/website selection, and account for such motivations in future comparative analyses.

The possible demographic differences between Jack’d and Grindr pose an additional limitation. By all reports, the ratio of White users to users of color is higher on Grindr than on Jack’d. Because each item on the scale describes an RSD experience as perpetrated by either a White or Black user, participants might have estimated frequency either in proportion to their total number of interactions or in proportion to their interactions with people of the race in question. For example, a participant who reports a low incidence of rejection from White men may be describing the effect of encountering relatively few White men overall or may be reporting on a lack of rejection from the White men they encounter. This uncertainty makes it difficult to distinguish between the effects of demographics and the effects of user culture in determining the prevalence of RSD and introduces an additional potential source of systematic error.

Despite the limitations noted above, this study has several notable strengths. It is among the first to attempt to contrast the prevalence of RSD on multiple platforms for YSMBM, and it does so using a psychometrically evaluated scale of RSD with a relatively large national sample. To the authors’ knowledge, this comparison study is unprecedented in the size of its sample and in the precision of its RSD measures. This study is also among the very few that have quantitatively compared different online dating environments for sexual minority men, and it contributes to an emerging area of study surrounding the nature of virtual environments and the experiences that marginalized people have within them.

## 7. Conclusions

Moving forward, it is important to examine online environments more closely and to examine RSD as it occurs between men of all races, and on a variety of other platforms. It will also be helpful to examine RSD using representative samples, and to compare online environments outside of the context of the United States. Different methods can expand on present understandings of the distinct user demographics and cultures of popular online dating environments. Because YSMBM are likely to use multiple apps and websites, researchers may consider asking study participants to directly compare and contrast their RSD-related experiences across different virtual environments. Researchers may also consider directly analyzing user profiles or creating dummy accounts on different apps/websites. A few studies have analyzed RSD in online environments by viewing large numbers of profiles [19,27,31,48] and future research can analyze racism in profiles using categories from the RSD scale. To gain insight into the role of RSD in the acceptance or rejection of potential partners and in personal messages, researchers can create dummy profiles to interact with real users. Robinson (2007) analyzed RSD on the website Adam4Adam.com using a version of this approach, in which they created profiles listing different races but presented the same torso photographs. Researchers can use similar approaches, such as adjusting the skin tones of pictures of models, to examine other aspects of RSD as it is practiced outside of user profiles, and to contrast the prevalence of these practices in different environments. Direct observation facilitates comparisons of digital spaces by treating the virtual environment itself as the unit of analysis.

Given that research in this area is underdeveloped, it will be important for investigators to use qualitative methods to explicate some of the nuances of cross-venue experiences that may be more difficult to ascertain using quantitative methods. More rigorous quantitative approaches are also needed—such as those described above—as well as cohort studies that directly assess the probability of encountering RSD, or of developing mental health complications related to RSD, based on exposure to different virtual environments. Overall, RSD remains a critical health issue, and there is considerable room to expand our current understanding of how different virtual environments may promote or impede RSD for sexual minority men of color.

## Figures and Tables

**Table 1:** Descriptive statistics for study sample.

Categorical Variables	*N* (*M*)	% (*SD*)			
Sexual Orientation					
Gay	387	70.6%			
Bisexual	89	16.2%			
Other	72	13.2%			
Education					
Less than high school	1	0.2%			
High school graduate	68	12.4%			
Some college	236	43.1%			
College graduate	155	28.3%			
Post college	88	16.1%			
Primary					
App Grindr	381	69.5%			
Jack’d	167	30.5%			
**Continuous Variables**	***M***	***SD***	**Min**	**Max**	**α**
Age	24.16	3.15	18	29	—
RSD Subscales					
White Rejection	57.50	25.44	0	100	0.931
Same-Race Rejection	42.52	15.74	0	100	0.886
White Superiority	42.02	18.73	0	96.43	0.823
White Physical Objectification	54.36	25.62	0	100	0.801
Same-Race Physical Objectification	45.63	23.65	0	100	0.772
Role Assumptions	41.70	22.16	0	100	0.833

**Table 2:** Pearson correlations among RSD subscales.

	1	2	3	4	5	6
1. White Rejection	–					
2. Same-Race Rejection	0.21 **	–				
3. White Superiority	0.46 **	0.22 **	–			
4. White Physical Objectification	0.11 *	0.21 **	0.33 **	–		
5. Same-Race Physical Objectification	−0.14 **	0.23 **	0.20 **	0.39 **	–	
6. Role Assumptions	0.13 **	0.16 **	0.48 **	0.37 **	0.33 **	–

* *p* < 0.05; ** *p* < 0.01.

**Table 3:** MKW test—mean rank differences on RSD by primary app.

	Grindr Users (*n* = 381)	Jack’d Users (*n* = 167)	*H*
White Rejection	304.87	205.79	47.85 ***
Same-Race Rejection	269.56	285.76	1.53
White Superiority	290.15	238.79	12.22 ***
White Physical Objectification	271.75	280.77	0.39
Same-Race Physical Objectification	247.38	336.37	38.00 ***
Role Assumptions	269.32	286.33	1.34

*** *p* < 0.001.

## Data Availability

The data presented in this study are available on request from the corresponding author.

## References

[1] Badal H.J., Stryker J.E., DeLuca N., Purcell D.W. (2018). Swipe right: Dating website and app use among men who have sex with men. AIDS Behav..

[2] Paz-Bailey G., Hoots B.E., Xia M., Finlayson T., Prejean J., Purcell D.W. (2017). Trends in internet use among men who have sex with men in the United States. J. Acquir. Immune Defic. Syndr..

[3] Vogels E.A. 10 Facts about Americans and Online Dating. Pew Research Center. https://www.pewresearch.org/fact-tank/2020/02/06/10-facts-about-americans-and-online-dating/.

[4] Grov C., Breslow A.S., Newcomb M.E., Rosenberger J.G., Bauermeister J. (2014). Gay and Bisexual Men’s Use of the Internet: Research from the 1990s through 2013. J. Sex Res..

[5] Gudelunas D. (2012). There’s an App for that: The Uses and Gratifications of Online Social Networks for Gay Men. Sex. Cult..

[6] Harper G.W., Serrano P.A., Bruce D., Bauermeister J.A. (2016). The Internet’s Multiple Roles in Facilitating the Sexual Orientation Identity Development of Gay and Bisexual Male Adolescents. Am. J. Men’s Health.

[7] Pingel E.S., Bauermeister J.A., Johns M.M., Eisenberg A., Leslie-Santana M. (2013). “A safe way to explore” reframing risk on the internet amidst young gay men’s search for identity. J. Adolesc. Res..

[8] Blackwell C., Birnholtz J., Abbott C. (2015). Seeing and being seen: Co-situation and impression formation using Grindr, a location-aware gay dating app. New Media Soc..

[9] Brown G., Maycock B., Burns S. (2005). Your picture is your bait: Use and meaning of cyberspace among gay men. J. Sex Res..

[10] Wade R.M., Harper G.W. (2020). Racialized sexual discrimination (RSD) in the age of online sexual networking Are gay/bisexual men of color at elevated risk for adverse psychological health?. Am. J. Community Psychol..

[11] Barlett C.P. (2015). Anonymously hurting others online: The effect of anonymity on cyberbullying frequency. Psychol. Pop. Media Cult..

[12] Conner C.T. (2019). The Gay Gayze: Expressions of Inequality on Grindr. Sociol. Q..

[13] Lauckner C., Truszczynski N., Lambert D., Kottamasu V., Meherally S., Schipani-McLaughlin A.M., Taylor E., Hansen N. (2019). “Catfishing,” cyberbullying, and coercion: An exploration of the risks associated with dating app use among rural sexual minority males. J. Gay Lesbian Ment. Health.

[14] Moore M.J., Nakano T., Enomoto A., Suda T. (2012). Anonymity and roles associated with aggressive posts in an online forum. Comput. Hum. Behav..

[15] Mowlabocus S., Ramos R., Mowlabocus S. (2020). A Kindr Grindr: Moderating race (ism) in techno-spaces of desire. Queer Sites in Global Contexts.

[16] Han C. (2008). No fats, femmes, or Asians: The utility of critical race theory in examining the role of gay stock stories in the marginalization of gay Asian men. Contemp. Justice Rev..

[17] Han C.S., Choi K.H. (2018). Very few people say “No Whites”: Gay men of color and the racial politics of desire. Sociol. Spectr..

[18] Paul J.P., Ayala G., Choi K.-H. (2010). Internet Sex Ads for MSM and Partner Selection Criteria: The Potency of Race/Ethnicity Online. J. Sex Res..

[19] Riggs D.W. (2013). Anti-Asian sentiment amongst a sample of white Australian men on Gaydar. Sex Roles.

[20] Coleman B.R., Collins C.R., Bonam C.M. (2021). Interrogating Whiteness in Community Research and Action. Am. J. Community Psychol..

[21] Sue D.W., Constantine M.G., Sue D.W. (2006). The invisible Whiteness of being: Whiteness, White supremacy, White privilege, and racism. Addressing Racism: Facilitating Cultural Competence in Mental Health and Educational Settings.

[22] Callander D., Holt M., Newman C. (2012). Just a preference: Racialised language in the sex-seeking profiles of gay and bisexual men. Cult. Health Sex..

[23] Callander D., Holt M., Newman C.E. (2016). ‘Not everyone’s gonna like me’: Accounting for race and racism in sex and dating web services for gay and bisexual men. Ethnicities.

[24] Callander D., Newman C.E., Holt M. (2015). Is Sexual Racism Really Racism? Distinguishing Attitudes Toward Sexual Racism and Generic Racism Among Gay and Bisexual Men. Arch. Sex. Behav..

[25] White J.M., Reisner S.L., Dunham E., Mimiaga M.J. (2014). Race-based sexual preferences in a sample of online profiles of urban men seeking sex with men. J. Urban Health.

[26] Cascalheira C.J., Smith B.A. (2020). Hierarchy of Desire: Partner Preferences and Social Identities of Men Who Have Sex with Men on Geosocial Networks. Sex. Cult..

[27] Robinson B.A. (2015). “Personal preference” as the new racism: Gay desire and racial cleansing in cyberspace. Sociol. Race Ethn..

[28] Robinson R.K. (2007). Structural dimensions of romantic preferences. Law Rev..

[29] Rosengren A.L., Menza T.W., LeGrand S., Muessig K.E., Bauermeister J.A., Hightow-Weidman L.B. (2019). Stigma and mobile app use among young black men who have sex with men. AIDS Educ. Prev..

[30] McKeown E., Nelson S., Anderson J., Low N., Elford J. (2010). Disclosure, discrimination and desire: Experiences of Black and South Asian gay men in Britain. Cult. Health Sex..

[31] Stacey L., Forbes T.D. (2021). Feeling Like a Fetish: Racialized Feelings, Fetishization, and the Contours of Sexual Racism on Gay Dating Apps. J. Sex Res..

[32] Wilson P.A., Valera P., Ventuneac A., Balan I., Rowe M., Carballo-Diéguez A. (2009). Race-based sexual stereotyping and sexual partnering among men who use the internet to identify other men for bareback sex. J. Sex Res..

[33] Calabrese S.K., Rosenberger J.G., Schick V.R., Novak D.S. (2015). Pleasure, Affection, and Love Among Black Men Who Have Sex with Men (MSM) versus MSM of Other Races: Countering Dehumanizing Stereotypes via Cross-Race Comparisons of Reported Sexual Experience at Last Sexual Event. Arch. Sex. Behav..

[34] Husbands W., Makoroka L., Walcott R., Adam B., George C., Remis R.S., Rourke S.B. (2013). Black gay men as sexual subjects: Race, racialisation and the social relations of sex among Black gay men in Toronto. Cult. Health Sex..

[35] Bedi S. (2015). Sexual Racism: Intimacy as a Matter of Justice. J. Politics.

[36] Daroya E., Riggs D.W. (2017). Erotic capital and the psychic life of racism on Grindr. The Psychic Life of Racism in Gay Men’s Communities.

[37] Wade R.M., Harper G.W. (2021). Toward a multidimensional construct of racialized sexual discrimination (RSD): Implications for scale development. Psychol. Sex. Orientat. Gend. Divers..

[38] Bhambhani Y., Flynn M.K., Kellum K.K., Wilson K.G. (2020). The Role of Psychological Flexibility as a Mediator Between Experienced Sexual Racism and Psychological Distress Among Men of Color Who Have Sex with Men. Arch. Sex. Behav..

[39] Brennan D.J., Asakura K., George C., Newman P.A., Giwa S., Hart T.A., Souleymanov R., Betancourt G. (2013). “Never reflected anywhere”: Body image among ethnoracialized gay and bisexual men. Body Image.

[40] English D., Hickson D.A., Callander D., Goodman M.S., Duncan D.T. (2020). Racial Discrimination, Sexual Partner Race/Ethnicity, and Depressive Symptoms Among Black Sexual Minority Men. Arch. Sex. Behav..

[41] Han C.-S., Ayala G., Paul J.P., Boylan R., Gregorich S.E., Choi K.-H. (2015). Stress and Coping with Racism and Their Role in Sexual Risk for HIV Among African American, Asian/Pacific Islander, and Latino Men Who Have Sex with Men. Arch. Sex. Behav..

[42] Hidalgo M.A., Layland E., Kubicek K., Kipke M. (2019). Sexual Racism, Psychological Symptoms, and Mindfulness Among Ethnically/Racially Diverse Young Men Who Have Sex with Men: A Moderation Analysis. Mindfulness.

[43] Meanley S., Bruce O., Hidalgo M.A., Bauermeister J.A. (2020). When young adult men who have sex with men seek partners online: Online discrimination and implications for mental health. Psychol. Sex. Orientat. Gend. Divers..

[44] Thai M., Stainer M.J., Barlow F.K. (2019). The “preference” paradox: Disclosing racial preferences in attraction is considered racist even by people who overtly claim it is not. J. Exp. Soc. Psychol..

[45] Wade R.M., Bouris A.M., Neilands T.B., Harper G.W. (2021). Racialized sexual discrimination (RSD) and psychological wellbeing among young sexual minority Black men (YSMBM) who seek intimate partners online. Sex. Res. Soc. Policy.

[46] Suler J. (2004). The online disinhibition effect. Cyberpsychol. Behav..

[47] Bonos L. What Is Jack’d? The Gay Dating App, Explained. The Washington Post.

[48] Chan L.S., Cassidy E., Rosenberger J.G. (2021). Mobile dating apps and racial preferencing insights: Exploring self-reported racial preferences and behavioral racial preferences among gay men using Jack’d. Int. J. Commun..

[49] Duncan D.T., Park S.H., Hambrick H.R., Ii D.T.D., Goedel W.C., Brewer R., Mgbako O., Lindsey J., Regan S.D., A Hickson D. (2018). Characterizing Geosocial-Networking App Use Among Young Black Men Who Have Sex With Men: A Multi-City Cross-Sectional Survey in the Southern United States. JMIR Mhealth Uhealth.

[50] Nahmod D.-E. Jack’d Goes after Grindr for Alleged Racism. The Bay Area Reporter. https://www.ebar.com/news/news//251703.

[51] Grindley L. Jack’d Attacks Grindr for Racist Profiles. The Advocate. https://www.advocate.com/business/2017/10/26/jackd-attacks-grindr-racist-profiles.

[52] Henry P. Dear White Gay Men, Racism Is not “Just a Preference.” Them. https://www.them.us/story/racism-is-not-a-preference.

[53] Guobadla O. Gay Communities are Rife with Racism. Removing Grindr’s Ethnicity Filters Won’t Fix That. GQ Magazine.

[54] West T. White Gays, Please Stop Including BBC in Your Grindr Profile. Medium. https://medium.com/queens-of-the-bs/white-gays-please-stop-including-bbc-in-your-grindr-profile-322705be2de0.

[55] Truong K. Asian-American Man Plans Lawsuit to Stop ‘Sexual Racism’ on Grindr. NBCUniversal News Group. https://www.nbcnews.com/feature/nbc-out/asian-american-man-threatens-class-action-discrimination-suit-against-grindr-n890946.

[56] Lang N. The Painful Reality of Sexual Racism in The Gay Community out Magazine. https://www.out.com/news-opinion/2017/5/15/truth-about-race-dating.

[57] Mastroyiannis A. Gay Dating Apps: A Comprehensive Guide to Jack’d, Grindr, Hornet, Scruff and the Rest. Pink News. https://www.pinknews.co.uk/2018/03/05/best-gay-dating-apps-jackd-grindr-hornet-scruff/.

[58] Bauermeister J.A., Pingel E., Zimmerman M., Couper M., Carballo-Dieguez A., Strecher V.J. (2012). Data quality in HIV/AIDS web-based surveys: Handling invalid and suspicious data. Field Methods.

[59] Fricker R.D., Fielding N., Lee R.M., Blank G. (2008). Sampling methods for web and e-mail surveys. The SAGE Handbook of Online Research Methods.

[60] Wade R.M., Harper G.W. (2021). Racialized sexual discrimination (RSD) in online sexual networking: Moving from discourse to measurement. J. Sex Res..

[61] Picardi P. Scruff CEO: The Real Issue with Grindr is Way Bigger than Gay Marriage. Out Magazine. https://www.out.com/out-exclusives/2018/12/03/scruff-ceo-eric-silverberg-responds-grindr-president.

[62] Business Wire Scruff Acquires Jack’d. Business Wire. https://www.businesswire.com/news/home/20190709005872/en/SCRUFF-Acquires-Jack%E2%80%99d.

[63] Winder T.J., Lea C.H. (2019). “Blocking” and “Filtering”: A commentary on mobile technology, racism, and the sexual networks of young black MSM (YBMSM). J. Racial Ethn. Health Disparities.

